# Satisfaction with Life, Subjective Well-Being and Functional Skills in Active Older Adults Based on Their Level of Physical Activity Practice

**DOI:** 10.3390/ijerph17041299

**Published:** 2020-02-18

**Authors:** María Antonia Parra-Rizo, Gema Sanchis-Soler

**Affiliations:** 1 Department of Health Psychology, Faculty of Social and Health Sciences, Campus of Elche, Miguel Hernandez University (UMH), 03202 Elche, Spain; 2 Department of General Didactics and Specific Didactics, University of Alicante, 03690 Alicante, Spain; gema.sanchis@ua.es

**Keywords:** physical activity, quality of life, satisfaction with life, subjective well-being, functional ability, functional autonomy, elderly

## Abstract

Studies about the influence of physical activity on life satisfaction, functional ability and subjective well-being in physically active older adults without cognitive impairment are very few for the moment. Therefore, the aim of this research was to evaluate the life satisfaction, functional skills and subjective well-being of physically active older adults based on the level of activity practiced. The IPAQ (International Physical Activity), CUBRECAVI and LSIA (Life Satisfaction Index) scales were tested for a sample of 397 Spanish older adults between 61 and 93 years of age (*M* = 69.65; *SD* = 4.71). The results showed that those who performed high physical activity obtained higher scores in functional skills (*p* < 0.01) and in the activities of daily living (*p* < 0.01). In addition, subjective well-being (*p* < 0.01) and the functional autonomy of older adults (*p* < 0.01) were related to the level of physical activity that they practiced. In conclusion, it could be said that the older adults with a high level of physical activity have more functional skills and less difficulties performing the activities of daily living, and that they value their autonomy and health better.

## 1. Introduction

The *2018–2020 Active and Healthy Aging Program* of the European Commission [1] emphasizes the need to implement policies that promote changes in the state of health and that show how to deal with chronic diseases in Europe. In order to achieve this, this Program points out that studies on the quality of life of the population of the European Union (EU) need to be carried out, as well as improvements in the provision of health-related services.

The World Health Organization (WHO), in its Global Action Plan on Physical Activity 2018–2030 [2], considers that people should remain active if they want to improve their present and future physical conditions.

Thus, international and national organizations associate physical exercise with health, especially in the situation of progressive aging that Europe has been facing for decades. Eurostat [3], for example, in its statistics on Demographic Structure and Aging of the European population, places the general aging rate in the continent at 19.4% and adds that the EU countries with the largest number of older adults are Germany, Italy, France, United Kingdom and Spain. On the other hand, some researchers [4] have emphasized that the Spanish longevity rate will continue to increase in the next decades by 10%; that is, one in three people will belong to the senior citizens segment. In addition, this author indicates that 6% of the Spanish population is over 80 years old. All these data can be explained because of the quality of life in Spain. Further, one of the factors that affects quality of life is physical exercise, which improves the dependency situations that are associated with age.

The Organization for Economic Cooperation and Development (OECD) [5], for its part, has considered this matter as “a public health problem” by stating that Spaniards live longer, but with more diseases and disabilities than their European neighbors, since their life expectancy, only behind Japan, means that 21% of older adults have limitations to carrying out their daily activities. This fact reflects the previously mentioned issue that there is a need to conduct studies into quality of life, healthy living and satisfaction with life during the old age.

In this regard, physical activity is one of the most important resources to improving health and quality of life in older adults, which has been revealed by different studies [6,7,8]. In addition to this, many authors have shown that physical activity improves the functional abilities of older adults [9,10,11] as well as their quality of life [12]. In a combined intervention study of physical activity and cognitive stimulation carried out in a sample of 50 older adults where 44 were women [13], an improvement in the functional abilities and activities of daily living were observed. Moreover, a recent review on this subject [14], concluded that combined strength and aerobic training causes improvements in the functional capacity and quality of life of older adults.

However, despite the demonstrated benefits that the practice of physical activity has on the quality of life [15], it is essential to consider the importance of the intensity and amount of physical activity that are carried out throughout the day and week. Thus, the World Health Organization recommends a practice of 150 min per week of moderate physical activity, or at least 75 min per week of intense physical activity. On the other hand, different authors have expressed a need to reduce the time dedicated to sedentary activities or low energy costs, since inactivity and sedentary lifestyles are related to a decrease in the quality of life of older adults [16,17].

Another aspect that has been approached in scientific literature is the relationship between type of physical exercise performed by older adults (low, moderate or high) and achieving better physical health combined with life satisfaction. Thus, [18] some researchers have discovered that only moderate physical activity (3–6 METs) is only related to physical health, while high activity (>6 METs) is related to life satisfaction as well as physical health. Above this, it has been observed that physical activity is also related to an improvement in functional abilities.

However, despite the research reviewed, [19] it has been pointed out that the perception of quality of life in physically active older adults seen from a multidimensional point of view has hardly been studied (that is to say, using instruments) [20,21,22] when combining diverse aspects such as health, social integration, functional skills, leisure activities, environmental quality, education, income and social and health services.

Regarding the need for scientific literature mentioned above, this study takes a look at physically active older adults in order to get to know if older adults with a high level of physical activity have better levels of well-being compared to older adults with low or moderate levels of physical activity. Specifically, the following issues need to be known:

(a) If there are differences between people with high engagement in physical activity with respect to practitioners with moderate or low physical activity in relation to life satisfaction.
(b) If there are differences between people with high engagement in physical activity compared to practitioners with moderate or low physical activity, in relation to subjective well-being and functional skills.

## 2. Method

### 2.1. Participants

The sample consisted of 397 older adults from the province of Alicante (Spain) with an average age of 69.65 years (*SD* = 4.71), where the minimum age was 61 years and the maximum was 93 years (72.3% were between 61 and 70 years of age, 23.7% were between 71 and 80 years of age and 4.0% were over 80 years of age). Of the sample, 64.7% were women and 35.3% were men. Regarding their marital status, 66% were married, 15.6% widowed, 7.8% single, 5.8% divorced and 4.8% had another situation. Of the participants, 31.6% lived alone.

Three inclusion criteria were established for the selection of participants: (a) they had to be 60 years old or above 60; (b) they had to be physically active; and (c) they had to have been practicing physical activity for more than one year. The exclusion criteria were: (a) anyone under 60 years of age; (b) older adults who showed that they were verbally inactive or sedentary, as well as who did not participate in physical activity; and (c) older adults with no ability to read the battery of questionnaires that needed to be answered.

Two aspects were analyzed in order to get to know the participants’ health conditions: lifestyle habits that could be considered unhealthy (such as consumption of tobacco and alcohol), and clinical status (physical and psychological diseases). Regarding their habits, 91.7% were non-smokers whereas 8.3% were smokers (4.3% smoked more than five cigarettes a day). In addition to this, 51.6% never consumed alcohol while 48.4% did consume alcohol (20.2% consumed daily, 21.1% sometimes during the week and 7.1% occasionally). Regarding clinical status, 71.3% did not suffer from any physical illness while 87.9% were not affected by psychological problems. Specifically, the physical ailments with the highest incidence were osteoarthritis (6.6%), hypertension (4.1%), diabetes (3%) and osteoporosis (2.8%), while the predominant psychological ailments were depression (8.5%) and anxiety (2.8%). It is possible that this low degree of disease, even among those most characteristic of adulthood (hypertension and diabetes), was due to the fact that these were physically active people.

### 2.2. Instruments

#### 2.2.1. International Physical Activity Questionnaire, IPAQ 

The Physical Activity Questionnaire [21] evaluates three types of physical activities: low intensity activity (walking), moderate intensity activities and high intensity activities. It also allows the classification of participants into three activity levels: high, moderate and low. These activity levels correspond to: <3 METs, 3–6 METs and >6 METs, respectively [23].

Participants with high activity levels are those who practice at least one more hour of moderate intensity activity daily above the baseline activity level, or half an hour of a high intensity activity above the baseline daily levels. Participants with moderate activity levels are those who practice at least half an hour of physical activity of moderate intensity almost every day. Participants with low activity levels are those who without moderate or high activity levels [24]. The short version of this questionnaire used in this study asked participants to answer seven items related to the physical activity that they carried out during the past seven days. The short version of the IPAQ has a reliability coefficient of 0.65 (*r_s_* = 0.76; 95% *CI*: 0.73–0.77). This questionnaire has recently been used in studies related to older adults [24,25].

#### 2.2.2. Brief Questionnaire on Quality of Life, CUBRECAVI 

This questionnaire consists of 21 subscales grouped into nine scales among which subjective health and functional skills (functional autonomy and activities of daily living) have been considered in order to carry out this study. This questionnaire is highly recommended to assess quality of life [16]. A participant must value the degree of satisfaction or the frequency of the different issues that are taken into account. The length of the questionnaire is approximately of 20 min. The levels of internal consistency of the scales range between 0.70 and 0.92. This questionnaire was used recently to assess quality of life in older adults [26].

#### 2.2.3. Life Satisfaction Index, Index A 

The Life Satisfaction Index [22] has five aspects: enthusiasm, purpose and strength, congruence between desire and the real possibility of reaching goals, positive self-concept and mood. The original LSIA scale consisted of 20 items, but was reviewed [22] and reduced to 18 items in which participants have to assess their level of conformity with score values ranging from 0 to 2 (0 = Disagree, 1 = Don’t know and 2 = Agree). The internal consistency level of the scale is α = 0.74. This scale has obtained good results when used with similar participants in other research studies [27].

### 2.3. Procedure

People in charge of 38 sports and cultural centers in Alicante were contacted in order to inform them about the ongoing study, then subsequently asked for their collaboration. In each of the 18 sports and cultural centers that agreed to collaborate, and after confirming that all the inclusion requirements were met, the interested attendees were given an informed consent form together with the questionnaire that they had to complete individually, as well as an explanation of the study and its goals.

After the participants performed their physical activities, they were delivered questionnaires in an envelope that had to be returned, once completed, in a follow-up appointment that was fixed at that time, guaranteeing the confidentiality of the data.

### 2.4. Analysis of Data

With the aim of knowing the characteristics of the sample, descriptive statistics of mean, minimum, maximum, frequency, percentage and standard deviation were performed. In order to study the differences between the participants according to their level of physical activity, a one-way ANOVA was applied for the quantitative variables while a chi-square test was used for the qualitative variables.

To estimate the size of the effect, an eta square (*ƞ^2^*) was used for the quantitative variables and a Phi coefficient and Cramer’s *V* were used (depending on the size of the contingency tables) for the qualitative variables.

The established significance value was <0.05.

Data analyses were performed with the SPSS statistical package, version 23.0 (IBM corp., Armonk, NY, USA for Windows).

## 3. Results

In order to know the differences in life satisfaction based on the level of physical activity practiced, a one-way ANOVA was performed. The results showed that the life-satisfaction scores of the participants who a high level of physical activity (23.16) were higher than those obtained by the participants who performed a moderate level (21.80) or low level (21.29) of physical activity; these differences, although close, were not statistically significant (*F*(2394) = 2977, *p* = 0.052, *ƞ*^2^ = 0.015) (Table 1).

Secondly, with the aim of knowing the differences in quality based on the level of physical activity practiced, the results showed that the scores of the participants in the functional skills scale varied according to the level of physical activity that they performed (*F*(2, 122.83) = 11.93, *p* < 0.001, *ƞ*^2^ = 0.054) (Table 2), and demonstrated the differences between those who performed a high level versus a moderate level of physical activity (3.79 and 3.59 respectively) (*p* < 0.001). There were no differences between the high and moderate levels a low level of physical activity, since being an active population, the percentage of people who performed a low level of activity was reduced compared to those who maintained a medium or high activity level.

In the subscale that evaluates the ability of participants to stand on their own (functional autonomy), statistically significant differences were found based on different levels of physical activity (χ^2^(4, *N* = 397) = 16.88; *p* = 0.002), with a small association between the variables (*V_Cramer_* = 0.146). In particular, there were significant differences found between those who had regular autonomy in comparison to others, as well as among those who performed a high level of activity with respect to others (χ^2^(1, *N* = 397) = 11.60; *p* = 0.001). There was a small association (*Phi* = 0.171) among of moderate and low activity participants with a degree of regular autonomy compared with those who demonstrated high physical activity (9.8% vs. 1.6%) (Table 3).

In the subscale of skills to perform daily activities it was noted that the scores of the participants varied according to the level of physical activity they performed (*F*(2, 117.55) = 8.62, *p* < 0.001, *ƞ^2^* = 0.043) (Table 4), focusing on the differences between those who performed a high level from those with a moderate level of physical activity (3.88 and 3.71 respectively) (*p =* 0.001) and among those who performed a high level and a low level of physical activity (3.88 and 3.62 respectively) (*p* = 0.047).

On the other hand, statistically significant differences were found regarding the degree of satisfaction with subjective well-being according to the level of physical activity of the participants (χ^2^(6, *N* = 397) = 33.58; *p* < 0.001), with a small association between variables (*V_Cramer_* = 0.206) (Table 5). Statistically significant differences in physical activity were found between the degree of no satisfaction and the other grades (χ^2^(2, *N* = 397) = 15.30; *p* < 0.001), with a small association between the variables (*V_Cramer_* = 0.196) revealing differences between those with a low activity and those with moderate and high activity (18.4% vs. 5.5%, respectively) (χ^2^(1, *N* = 397) = 10.92; *p* = 0.001; *Phi* = 0.166) and among those with high activity in comparison to those with moderate or low activity (2.7% vs. 10.7%, respectively) (χ^2^(1, *N* = 397) = 9.67; *p* = 0.002; *Phi* = 0.156). Statistically significant differences in physical activity were also found between degree of satisfaction and the other grades (χ^2^(2, *N* = 397) = 24.86; *p* < 0.001), with a small association between variables (*V_Cramer_* = 0.250) found in the differences between those with low activity and those with moderate and high activity (28.6% vs. 53.2%, respectively) (χ^2^(1, *N* = 397) = 10.39; *p* = 0.001; *Phi* = −0.162), between those with moderate activity and those with high and low activity (42.4% vs. 55.6%, respectively) (χ^2^(1, *N* = 397) = 6.70; *p* = 0.010; *Phi* = 0.130) and between those with high activity with those with moderate and low activity (62.8% vs. 39.3%, respectively) (χ^2^(1, *N* = 397) = 21.96; *p* < 0.001; *Phi* = −0.235).

## 4. Discussion

The aim of this research was to determine the satisfaction with life, functional abilities and perception of subjective health in physically active older adults without cognitive impairment based on their level of physical activity (high, moderate or low). Firstly, the results showed that although higher levels of satisfaction with life were related to physically active older adults who practice a high level of physical activity in comparison with those who perform moderate physical activity and those who perform low physical activity, the differences were not become significant. This result differed from the those obtained in other studies [28]. However, it is necessary to specify that there has been little research into life satisfaction related to level of physical practice in active older adults, as occurred in this study.

Secondly, in this study, functional ability was determined to be greater in those people who engaged in high physical activity compared to those who participated in moderate activity. These results are in line with the existing literature showing an improvement in physical health regarding functional skills in those older adults who practiced high physical activity [8,9,10,11]. On the other hand, some studies [29] have also indicated that there is a relationship between low autonomy and poor physical activity; however, some of these studies have not gone far enough into the amount and type of physical practice required for such a result. Other research has highlighted the importance of the type of activity performed as well as the intensity of the activity. In relation to the type of activity, different studies have pointed out the correlations among strength training, increased autonomy and improved quality of life [30,31]. Others have discussed the need to include aerobic training in exercise routines, as it improves cardiorespiratory capacity [32]. Finally, there are many who support the concept that combined physical training (strength, aerobic, flexibility, balance, etc.) will improve autonomy, functional capacity and quality of life of older adults [33,34,35].

In relation to intensity, some authors have shown that improvement in functional capacity takes place both in the practice of moderate activity and in the practice of high activity [18,36]. In this regard, some studies [37] have also refuted that the practice of low physical activity is ineffective for the functional improvement of older adults. These results have also been supported by other researchers [38] who obtained greater improvements in a HIIT-(High Intensity Interval Training) trained group than a group treated through regular low intensity continuous training.

Regarding the development of daily activities, the results of this study showed better scores for those individuals with higher levels of physical activity. As with the previous variables investigated here, there are a few studies that have been carried out on active older adults without physical deterioration or pathology. Previous works confirmed an improvement in performance of daily activities by the older adults studied, but their results were not separated by intensity [13].

In relation to the better perception of the subjective well-being achieved when high physical activity is practiced, some authors have considered that health along with functional independence are essential to facilitate positive self-perception as individuals age [39,40]. In fact, different studies have highlighted the need to work on the self-perception of health during the practice of physical exercise [41]. Our results, on the other hand, were not conclusive in this regard, possibly because it was a cross-sectional study.

It is necessary to point out that most studies in the area of old age have focused on cognitive impairment [42,43], strength and agility [44], or on the pathological perspective of old age (e.g., Alzheimer’s) [45]. Hence, there are few scientific studies at present that have focused on the positive promotion of health as well as other profiles without pathologies. It seems important to emphasize the promotion of health in healthy older adults. A lack of physical activity adds to the natural changes of aging, resulting in a large number of problems at physiological, psychological and physical levels such as hip fractures, depression, solitude and fragility, among others. All of these factors increase the risk of institutionalization [46,47,48,49].

This study shows that with an intense practice of physical activity, better psychophysical health was recorded; in other words, institutionalization can be avoided [50]. The present investigation, which evaluated physically active older adults, demonstrated a way to prevent the loss of health and functional capacities that are so related to the institutionalization of an increasingly large population internationally—and in Spain, in particular [4].

Regarding future research, it would be interesting to know how enthusiasm, purpose and strength in life satisfaction are related to different levels of physical practice, as well as to their relationships with other aspects of quality of life concerning health. It would also be appropriate to study the profiles of those who practice intense physical activity in particular, as well as to study individuals who practice moderate and low physical activity. In this way, we could elucidate which specific aspects encourage greater physical practice and, consequently, how to design more effective programs to promote health.

Among the main limitations of this research work, it is worth highlighting that with this sample (physically active older adults) it was not possible to extrapolate the results obtained. Therefore, more studies with these characteristics are needed. Likewise, the practice of physical activity was measured through a self-report, the IPAQ [21], and the use of more objective measures would be highly recommended in future works.

## 5. Conclusions

Given the lack of studies in the scientific literature on physically active older adults without deterioration or pathologies, this cross-sectional research shows that a high amount of physical activity improves functional skills in old age (autonomy and ability to perform activities of daily living) compared with practicing moderate or low physical activity. This finding shows that the practice of high physical activity should be included in health promotion programs in order to avoid functional dependence. On the other hand, more longitudinal studies are required of older adults, to confirm the relationship between level of physical activity practiced and life satisfaction. 

## Figures and Tables

**Table 1:** Means, standard deviations and one-way ANOVA of the participants’ scores on the life satisfaction scale (LSIA) based on their level of physical activity.

LSIA	High Ph.A.		Moderate Ph.A.		Low Ph.A.		*F*	*p*	*ƞ^2^*
	*n*	*M* (*SD*)	*n*	*M* (*SD*)	*n*	*M* (*SD*)			
**Life satisfaction**	183	23.16 (5.76)	165	21.80 (6.31)	49	21.29 (7.121)	2.977	0.052	0.015

*Note n* = number of participants; *M* = Mean; *SD* = Standard deviation *F* = statistic F; *p* = *p*-value; *ƞ*^2^ = eta square effect size, Ph.A. = Physical Activity.

**Table 2:** Means, standard deviations and one-way ANOVA of the participant scores in the functional skills scale (CUBRECAVI) according to their level of physical activity.

Functional Skills Scale	High Ph.A.		Moderate Ph.A		Low Ph.A.		*F*	*p*	*ƞ^2^*
	n	M (SD)	n	M (SD)	n	M (SD)			
**Functional skills**	183	3.79 (0.34)	165	3.59 (0.46)	49	3.63 (0.52)	11.926	0.000 *	0.054

*Note* * *p* < 0.05; *n* = number of participants; *M* = Mean; *SD* = Standard deviation *F* = statistic F; *p* = *p*-value; *ƞ*^2^ = eta square effect size.

**Table 3:** Frequencies of the functional autonomy subscale (CUBRECAVI) according to level of physical activity.

Level of Physical Activity	Bad		Regular		Good		Very Good	
	*n*	*%*	*n*	*%*	*n*	*%*	*n*	*%*
High Ph.A	0	0.0	3	1.6	48	26.2	132	72.1
Moderate.	0	0.0	18	10.9	53	32.1	94	57.0
Low Ph.A	0	0.0	3	6.1	12	24.5	34	69.4

*n* = number of participants; % = percentage.

**Table 4:** Means, typical deviations and one-way ANOVA of the participant scores in the subscale of activities of daily living (CUBRECAVI) according to their level of physical activity.

Activities of Daily Living Scale	High Ph.A.		Moderate Ph.A		Low Ph.A.		*F*	*p*	*ƞ^2^*
	n	M (SD)	N	M (SD)	n	M (SD)			
Daily activities	183	3.88 (0.36)	165	3.71 (0.48)	49	3.62 (0.72)	8.618	0.000 *	0.043

*Note* * *p* < 0.05. *n* = number of participants; *M* = Mean; *SD* = Standard deviation *F* = statistic F; *p* = p-value; *ƞ*^2^ = eta square effect size.

**Table 5:** Frequencies of the subjective health scale (CUBRECAVI) according to level of physical activity.

	No Satisfied at All		A Little Satisfied		Fairly Satisfied		Very Satisfied	
	*n*	*%*	*n*	*%*	*n*	*%*	*n*	*%*
High Ph.A.	5	2.8	39	21.3	115	62.8	24	13.1
Moderate	14	8.5	48	29.1	70	42.4	33	20.0
Low Ph.A	9	18.4	13	26.5	14	28.6	13	26.5

*n* = number of participants; % = percentage.
